# Microbial fertilizer for improving maize yield, straw decomposition and soil microbiome structure

**DOI:** 10.3389/fmicb.2025.1670118

**Published:** 2025-12-15

**Authors:** Xianjin Xie, Hua Tao, Yanan Li, Xueying Feng, Ke Li, Zhehui Zhang, Junying Yan, Xiaolin Wang

**Affiliations:** 1 Henan Soil and Fertilizer Station, Zhengzhou, Henan, China; 2 University of Chinese Academy of Sciences, Beijing, China

**Keywords:** microbial fertilizer, soil microbiome, maize yield, straw decomposition, nutrient cycling, agroecological zones

## Abstract

Microbial fertilizers represent a promising strategy to sustainably produce crops by enhancing the biological function of soil and availability of nutrients. However, there is a lack of study on their performance across diverse agroecological zones. In this study, we conducted a 3-year, two-site field experiment to assess the effects of a composite microbial fertilizer (*Bacillus subtilis* and *Trichoderma harzianum*) on the yield of maize (*Zea mays*), soil properties, straw degradation, and composition of the microbial community. The results showed that the microbial fertilizer treatment (MF) increased the yield of maize by 11.4 and 6.9% in Qingfeng Country (QF) and Xun Country (Xun), China, respectively, compared to normal chemical fertilizer (CF). These gains coincided with an enhanced straw degradation rate (SDR; +8.4–8.6%) and a tendency toward higher available phosphorus (AP; +15.4–19.7%), alongside shifts in bacterial and fungal composition. High-throughput sequencing revealed that Proteobacteria, Actinobacteriota, Acidobacteriota, and Chloroflexi dominated the bacterial communities at both sites, whereas the fungal communities were mainly composed of Sordariomycetes, Dothideomycetes, and Eurotiomycetes—taxa whose abundances displayed pronounced site specificity. Application of the microbial fertilizer was associated with higher relative abundance of Acidobacteriota by 22.7% (QF) and 60.8% (Xun) and that of Sordariomycetes by 13.7% (QF) and 30.9% (Xun), underscoring its strong, selective impact on the dominant bacterial and fungal assemblages. These regional differences underscore the influence of site-specific microbial assemblages on the performance of fertilizer. Partial least squares path modeling supported a plausible pathway in which changes in community structure and straw decomposition are linked to improved soil nutrient status, which in turn predicted yield (β = 0.846, *R*^2^ = 0.715). Together, the field data indicate that microbial fertilizers may act through multi-step, microbiome-associated pathways, with success depending on compatibility with native microbial assemblages and environmental context.

## Highlights

- Composite microbial fertilizers improved yield > 10% across 2 agroecological zones.
- The microbial community structure and function drove the enhanced yield benefits.
- Microbial fertilizer significantly increased the soil available phosphorus.
- In addition, microbial fertilizer increased the decomposition of straw.
- SEM revealed a multi-step, biologically mediated yield enhancement pathway.

## Introduction

The application of microbial fertilizers (microbial inoculants) has emerged as a pivotal strategy to enhance the sustainability of agriculture by improving soil health, nutrient cycling, and crop productivity (O’Callaghan et al., 2022; Wang T. et al., 2024). These inoculants, which are composed of bacteria, fungi, and other microorganisms, are designed to perform key functions, such as nitrogen fixation, phosphorus solubilization, and the decomposition of organic matter, and offer sustainable alternatives to synthetic fertilizers and pesticides (Kaminsky et al., 2019). However, their ecological impacts remain context-dependent since they are shaped by the microbial community dynamics in the soil, environmental conditions, and regional edaphic factors (Mawarda et al., 2020; Kong et al., 2025). The introduction of exogenous microbial consortia can induce both transient and persistent shifts in the assembly of the soil microbiome, with cascading effects on the mobilization of nutrient dynamics, pathogen antagonism, and rhizosphere signaling networks (Trabelsi and Mhamdi, 2013; O’Callaghan et al., 2022). Crucially, the legacy effects of such interventions are contingent upon the compatibility between inoculants and indigenous microbial guilds. This is a factor that is often overlooked in cross-regional studies.

In maize (*Zea mays*) production systems, microbial fertilizers offer promise to optimize the yield and efficiency of nutrient use under intensive farming practices (Liu et al., 2022; Shahwar et al., 2023). *Bacillus subtilis* (*B. subtilis*) and *Trichoderma harzianum* are microorganisms that are widely used as fertilizers. They are known for promoting plant growth and suppressing plant pathogens through a variety of mechanisms, including the solubilization of nutrients, production of phytohormones, and their enhancement of induced systemic resistance (Blake et al., 2021; Joo and Hussein, 2022; Guzmán-Guzmán et al., 2023; Wang J. et al., 2024; Petkova et al., 2025). Their synergistic application offers a sustainable alternative to chemical fertilizers and pesticides and enhances both crop productivity and soil health. Nevertheless, there has been little study on the extent to which these inoculants reshape the structure-function relationships of microorganisms in the soil and persist over time. Priority effects—where early-colonizing microbes influence the subsequent community assembly (Debray et al., 2022)—play a critical role in the success of inoculants through mechanisms, such as niche preemption, facilitation, or inhibition (Kong et al., 2025). Additionally, root exudates, modulated by plant growth-promoting microorganisms, further mediate the microbial interactions in the rhizosphere and highlight both direct and indirect pathways through which inoculants exert their effects (Dardanelli et al., 2010; Walker et al., 2011).

The incorporation of straw is a common practice to recycle crop residues. However, its decomposition under field conditions is often slow, which can lead to issues, such as hindered tillage and the immobilization of nutrients (Kuang et al., 2014). Integrating microbial decomposers into the management of straw has been shown to enhance its breakdown, increase the content of dissolved organic carbon, and improve subsequent crop yields (Wang et al., 2020; Zhao et al., 2024). However, few current studies have compared and evaluated the straw degradation rate (SDR), soil nutrient dynamics, succession of microbial community structure, and the growth of crops across different agroecological zones through the application of compound microbial inoculants or fertilizers.

To address existing knowledge gaps, we conducted a two-way factorial field experiment at two ecologically distinct sites, comparing standard chemical fertilization with a combined application of chemical and microbial fertilizers. Key measurements included soil available phosphorus (AP), total nitrogen (TN), soil organic carbon (SOC), SDR, maize yield, and the composition and diversity of the soil microbial community. Structural equation modeling (SEM) was utilized to unravel the direct and indirect pathways that link microbial fertilization to crop yield. This approach provides valuable insights for developing region-specific microbial fertilization strategies. These contrasts provided a valuable context to test the consistency and mechanisms of the performance of microbial fertilizers under varying ecological conditions. The contrasting environmental conditions across the two sites provided a robust framework to evaluate the consistency and underlying mechanisms of microbial fertilizer performance. It was hypothesized that microbial fertilizers would enhance maize yield by restructuring microbial communities and improving nutrient cycling, with the magnitude and mechanisms of these effects expected to vary according to local microbiome and nutrient dynamics.

## Materials and methods

### Experimental design

The field experiment was conducted continuously from 2018 to 2020 at two sites in Henan Province, China, within the Huang–Huai–Hai Plain: Wangzhuang Village, Gaobao Township, Qingfeng Country (QF; 35.9301°N, 115.1407°E) and Fuzhuang Village, Weixi Subdistrict Office, Xun Country (Xun; 35.6378°N, 114.4811°E). The sites, separated by approximately 86 km, are both warm-temperate, semi-humid monsoon climates with four distinct seasons. QF exhibits an annual mean temperature of 13.4°C, mean annual precipitation of 540 mm, and a frost-free period of approximately 215 days. In contrast, Xun has a slightly higher mean temperature (13.7°C), greater precipitation (∼648 mm), and an extended frost-free period (∼221 days), which is indicative of a more humid, temperate microclimate. Both sites feature flat terrain, a double-cropping system, and a long-term wheat (*Triticum aestivum*)–maize rotation.

The soil at the QF site is classified as calcareous fluvo-aquic soil, with pre-experiment soil properties (0–20 cm depth) as follows: SOC, 8.53 g/kg; TN, 0.94 g/kg; AP, 19.8 mg/kg; available potassium (AK) 10^4^, mg/kg; slowly available K (SAK), 1,019 mg/kg; pH, 8.2; and the soil texture is light loam. The Xun site features alkaline fluvo-aquic soil with the following pre-experiment soil properties: SOC, 13.9 g/kg; TN, 1.39 g/kg; AP, 21.8 mg/kg; AK, 369 mg/kg; and SAK, 1,023 mg/kg; pH, 8.7. The soil texture is heavy clay.

Two treatments were established as follows: CF (chemical fertilizer) and MF (chemical fertilizer combined with microbial fertilizer). Both treatments received identical rates of N (210 kg/ha), phosphorus pentoxide (P_2_O_5_) (45 kg/ha), and potassium oxide (K_2_O) (45 kg/ha). The microbial fertilizer (75 kg/ha) contained *B. subtilis* (3.0 × 10^8^ CFU/g) and *Trichoderma harzianum* (4.0 × 10^6^ CFU/g) embedded in a clay-humic acid carrier. Nitrogen was applied in two splits (basal: topdressing = 11:3, w/w), while P and K were applied as basal fertilizers. The microbial fertilizer was mixed with basal fertilizers and applied using a deep-loosening combined seeding-fertilization machine (depth: 25–30 cm; fertilization depth: 8–15 cm; 2BMSQFY, Hebei Nonghaha Machinery Group Co., Ltd., Heibei Province, China). There were three replicates for each treatment plot (6 × 100 m). The maize varieties used were Zhengdan 958 (QF) and Xundan 29 (Xun), which are the regionally recommended maize varieties. The previous crop was wheat, and the wheat straw was retained and incorporated into the field. Other management practices were carried out according to local farmer conventions, with consistent implementation across all treatments.

### Straw degradation rate

In the third year of the study, nylon bags that contained wheat straw were buried in each treatment plot to monitor the amount of wheat straw that remained as residue. The wheat straw was chopped into 2–3 cm pieces, and 15 g (oven-dried weight) was placed into nylon bags with a pore size of 180 μm. The maize was sown by burying the bags approximately 10 cm deep in the soil. Five nylon bags that contained the chopped straw were evenly and equidistantly buried within each subplot. The maize was harvested by retrieving the bags and carefully washing them on a 0.25-mm sieve to remove the soil particles. The samples were then oven-dried and weighed to determine the remaining straw residue.

### Maize yield and soil sampling

Plant samples were collected at maize maturity in each of three consecutive years. At physiological maturity, 20 consecutive plants were sampled from a randomly selected position within the central row. Ears were immediately husked and shelled; fresh grain mass was determined on a calibrated bench scale. A representative subsample was oven-dried at 65°C to constant weight for moisture determination. Stand density was quantified from plant counts along a measured row length of known spacing, and calculated the yield for the total plants per hectare.

Soil samples were collected from three replicate plots of each treatment at the third year of the study when the maize was harvested. Five soil samples were randomly collected from each plot and all the samples were immediately transported to the laboratory on ice. The soil samples of each plot were thoroughly mixed, passed through a 2-mm sieve. Each soil sample was divided into two subsamples. One was stored at −80°C for soil microbial community, and the other was air-dried to determine the soil physicochemical properties.

### Determination of soil physicochemical properties

Air-dried soil samples were utilized for the analysis of physicochemical properties. The SOC was determined via the potassium dichromate-sulfuric acid oxidation method, the TN via the Kjeldahl method, and the AP via the sodium bicarbonate extraction-molybdenum antimony colorimetric method.

### Soil DNA extraction and high-throughput sequencing

The soil DNA was extracted using the FastDNA^®^ Spin Kit for Soil (MP Biomedicals, Irvine, CA, United States). The V3–V4 region of the 16S rRNA (primers 338F/806R) (Yang et al., 2019) and ITS1 region (primers ITS1F/ITS2R) (Li et al., 2016) were amplified and sequenced on an Illumina MiSeq platform (Illumina, San Diego, CA, United States). The sequences were processed using fastp (Chen et al., 2018), FLASH (Magoč and Salzberg, 2011), and UPARSE (Edgar, 2013) (97% similarity threshold). The taxonomy was annotated against the SILVA 138 (16S) and UNITE 8.0 (ITS) databases (Wang et al., 2007). All raw FASTQ files and associated metadata have been deposited in NCBI SRA under BioProject PRJNA1331835.

### Quantitative PCR (qPCR)

The DNA extracts were analyzed for the abundance of *B. subtilis* using the primers Bsub_16S_F (5′-TCTGCTCGTGAACGGTGCT-3′) and Bsub_16S_R (5′-TTTCGCCTTATTTACTTGG-3′). The reactions contained 10 μL SYBR Green Mix, 1 μL DNA template, and 0.5 μM primers. The cycling conditions were as follows: 95°C for 3 min, 40 cycles of 95°C for 15 s and 60°C for 30 s. The copy numbers were calculated from a standard curve of plasmid DNA that contained the target gene. qPCR assays targeting the ITS region of Trichoderma harzianum were conducted using the same DNA extracts. Assay sensitivity in soil matrices reported for Trichoderma ranges on the order of ∼10^4^ conidia-equivalents g^−1^ soil, depending on the primer/probe set and matrix effects. Accordingly, values below this threshold were treated as below the method detection limit (MDL) and denoted as non-detects.

### Statistical analysis

A two-way ANOVA was conducted for SOC, TN, AP, SDR, SOC:TN (CNratio) and SOC:AP (CPratio) to determine the main and interactive effects of the treatment and location. A one-way analysis of variance (ANOVA) was conducted to assess the effects of treatment, year, and their interactions on yield. *Post-hoc* multiple comparisons were performed using Tukey’s honestly significant difference (HSD) test. The alpha-diversity indices (Shannon, Simpson, Richness, Chao1, and ACE) were calculated using the vegan package in R (version 4.5.1, R Core Team, Vienna, Austria). These indices were computed separately for the bacteria and fungi and grouped by site and treatment. The descriptive statistics and ANOVA were performed as above. *T*-tests were conducted within each location to assess the treatment effects for each index, and the results were summarized with their corresponding significance. The Mantel test was performed with the R package linkET to evaluate the correlations between the microbial community matrices (bacteria, fungi, and key OTUs) and environmental factors. The Mantel test results were superimposed on the correlation heatmap, with line color and thickness indicating the strength and significance of the correlations, respectively.

The co-occurrence networks were constructed based on pairwise Spearman correlations among the OTUs, with multiple-testing correction (Benjamini-Hochberg) implemented via psych::corr.test. Significant (adjusted *p* < 0.05) and strong (|r| > 0.6) associations were used to construct undirected, weighted networks using the igraph package in R. Network topology parameters, including the node number, edge number, average degree, clustering coefficient, density, modularity, and proportion of positive/negative correlations, were computed for each network. The networks were visualized in Gephi following their export in the GraphML format.

The keystone taxa within the co-occurrence networks were identified using the Zi-Pi method (Guimerà and Nunes Amaral, 2005). The within-module degree (Zi) and participation coefficient (Pi) were calculated for each node. The nodes were then classified as module hubs (Zi > 2.5, Pi < 0.62), connectors (Zi < 2.5, Pi > 0.62), network hubs (Zi > 2.5, Pi > 0.62), or peripherals (Zi < 2.5, Pi < 0.62).

We inferred putative bacterial functions from the 16S ASV table using PICRUSt2 (KEGG Orthologs/Enzymes and MetaCyc L3 pathways) and, for robustness, Tax4Fun (KEGG Orthologs/Enzymes). Features related to cellulose/hemicellulose degradation and phosphorus cycling were selected a priori with keyword/EC filters (full mapping in Supplementary Data 1, 2). Fungal trophic modes/guilds were annotated from ITS assignments using FUNGuild. All functional matrices were harmonized to the sample metadata and analyzed within each site. MF vs. CF differences were tested using ALDEx2 with Benjamini–Hochberg FDR adjustment. For trait associations, functional features were CLR-transformed and related to SDR and AP with Spearman correlations (FDR-adjusted). Visualization combines (i) bubble plots of effect size (MF−CF) versus correlation with SDR/AP and (ii) a guild–trait heatmap; significance is annotated with FDR where applicable.

The direct and indirect relationships among the management practices, microbial properties, soil variables, and crop yield were explored with the partial least squares path modeling (PLS-PM) using the plspm package in R. All the variables were standardized before they were analyzed. Latent variables and their reflective blocks (outer model) were: (i) Microbial (bacterial Shannon, fungal Simpson, keystone taxa abundance); (ii) SDR (straw decomposition rate); (iii) SoilNutrient (SOC, TN, AP, CPratio); (iv) Yield; and a grouping/control construct (Group). The inner (structural) model specified the following paths: Group ield; and a grouping/contrDR and SoilNutrient; SDR →RSoilNutrient; and SoilNutrient oilNutri The latent variables were constructed to aggregate the relevant observed indicators. Model significance and path coefficients were assessed via bootstrapping (500 resamples). We report standardized path coefficients, R^2^ of endogenous constructs, composite reliability and AVE/communality; plotting functions of plspm were used to visualize the path diagram.

## Results

### Effects of the site and microbial fertilizer on the soil properties, straw degradation, maize yield, and soil microbial community α-diversity

**FIGURE 1:**
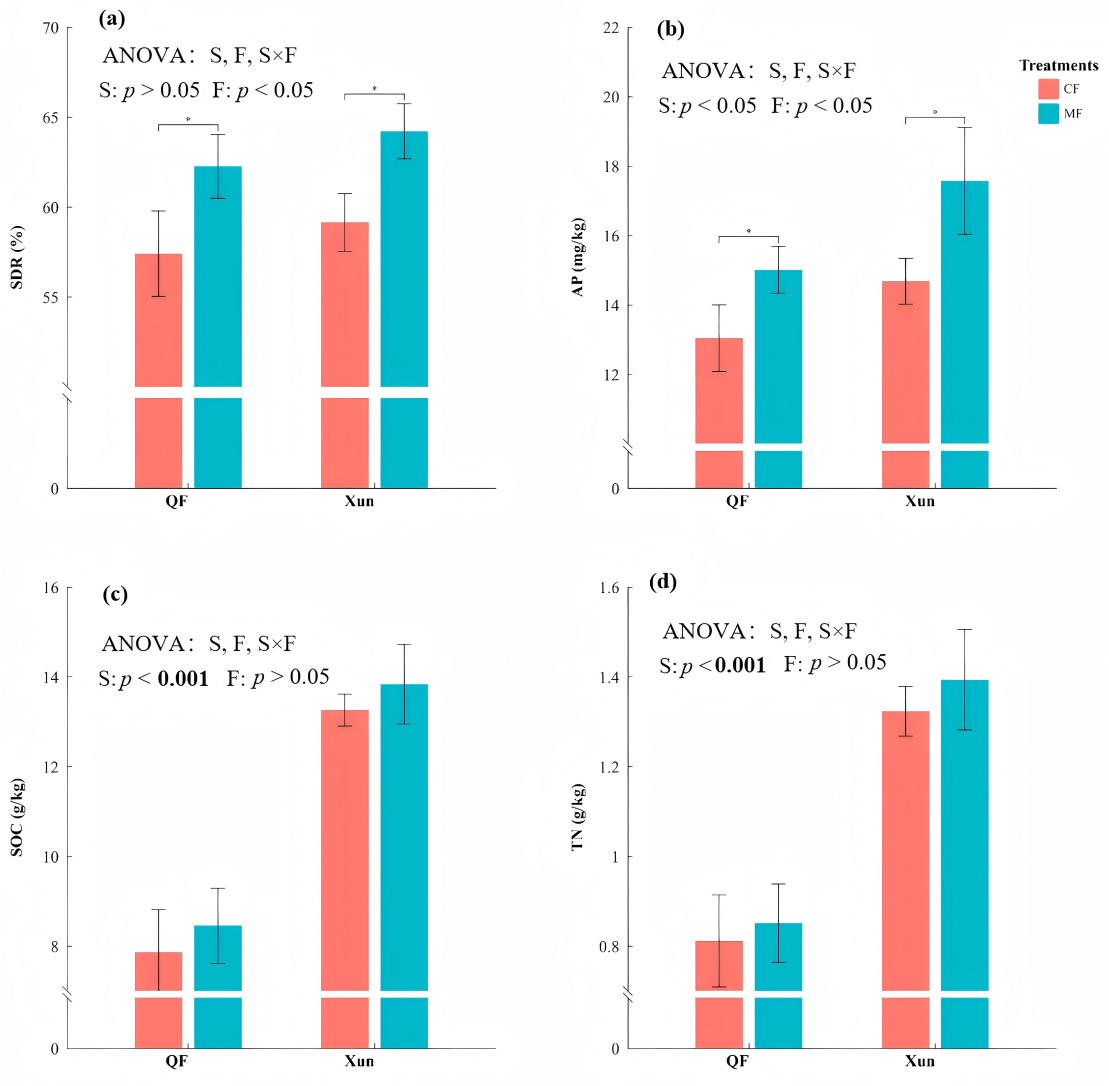
Comparative effects of chemical fertilization (CF) and microbial fertilizer (MF) on straw degradation and soil properties across two sites of QF and Xun. Bars show means ± SE for four response soil variables. (A) Straw degradation rate (SDR), (B) available phosphorus (AP, mg kg^−1^), (C) soil organic carbon (SOC, g kg^−1^), and (D) total nitrogen (TN, g kg^−1^) between the CF and MF treatments in two sites. ANOVA used to test the effects of sites (S), fertilization (F). *p < 0.05.

The two-way ANOVA results indicated significant effects of both the site and microbial fertilizer treatment on various soil and crop parameters (Figure 1 and Table 1). Soil AP showed significant main effects of site (*P* = 0.008) and fertilization (*P* = 0.004). Compared to the CF treatment, AP level in the MF treatment increased by 15.4% at QF (*p* < 0.05) and by 19.7% at Xun (*p* < 0.001) (Figure 1B). The TN was significantly affected by site (*p* < 0.001), with higher values observed at Xun. Although the microbial fertilizer slightly increased the content of TN by 4.9% at QF and 5.3% at Xun, these increases were not statistically significant (Figure 1D). The SOC also exhibited a significant site effect (*p* < 0.001). The SOC increased by 8.1% at QF and 3.9% at Xun following the application of microbial fertilizer (Figure 1C). At Xun, the CP ratio under MF was significantly lower than under CF (*P* < 0.01); QF showed no such difference. Across both sites, the C:N ratio remained unaffected by treatment (Supplementary Figure 1).

**TABLE 1:** Two-way ANOVA results to assess the effects of the application of microbial fertilizer and site on soil microbial α-diversity of bacteria and fungi.

Indicator	QF_CF	QF_MF	Xun_CF	Xun_MF	Site *p*-value	Treatment *p*-value	Interaction *p*-value
B_Richness	2690 ± 75.2	2740 ± 54.6	2570 ± 93.4	2580 ± 56.0	**0.011**	0.534	0.700
B_Shannon	6.63 ± 0.133	6.72 ± 0.0241	6.64 ± 0.0859	6.65 ± 0.0278	0.544	0.262	0.392
F_Richness	90.7 ± 18.0	96.0 ± 6.24	114 ± 2.52	115 ± 9.87	**0.009**	0.590	0.776
F_Shannon	2.92 ± 0.010	2.70 ± 0.111	2.45 ± 0.103	2.54 ± 0.565	0.096	0.716	0.392

Data are presented as mean ± standard error. QF, Qingfeng Country; Xun, Xun Country; CF, chemical fertilizer; MF, microbial fertilizer; B_Richness, bacterial species richness; F_Shannon, fungal Shannon index. Bold *p*-values indicate statistically significant main effects (Site, Treatment, or their Interaction) based on the two-way ANOVA (*p* < 0.05).

**FIGURE 2:**
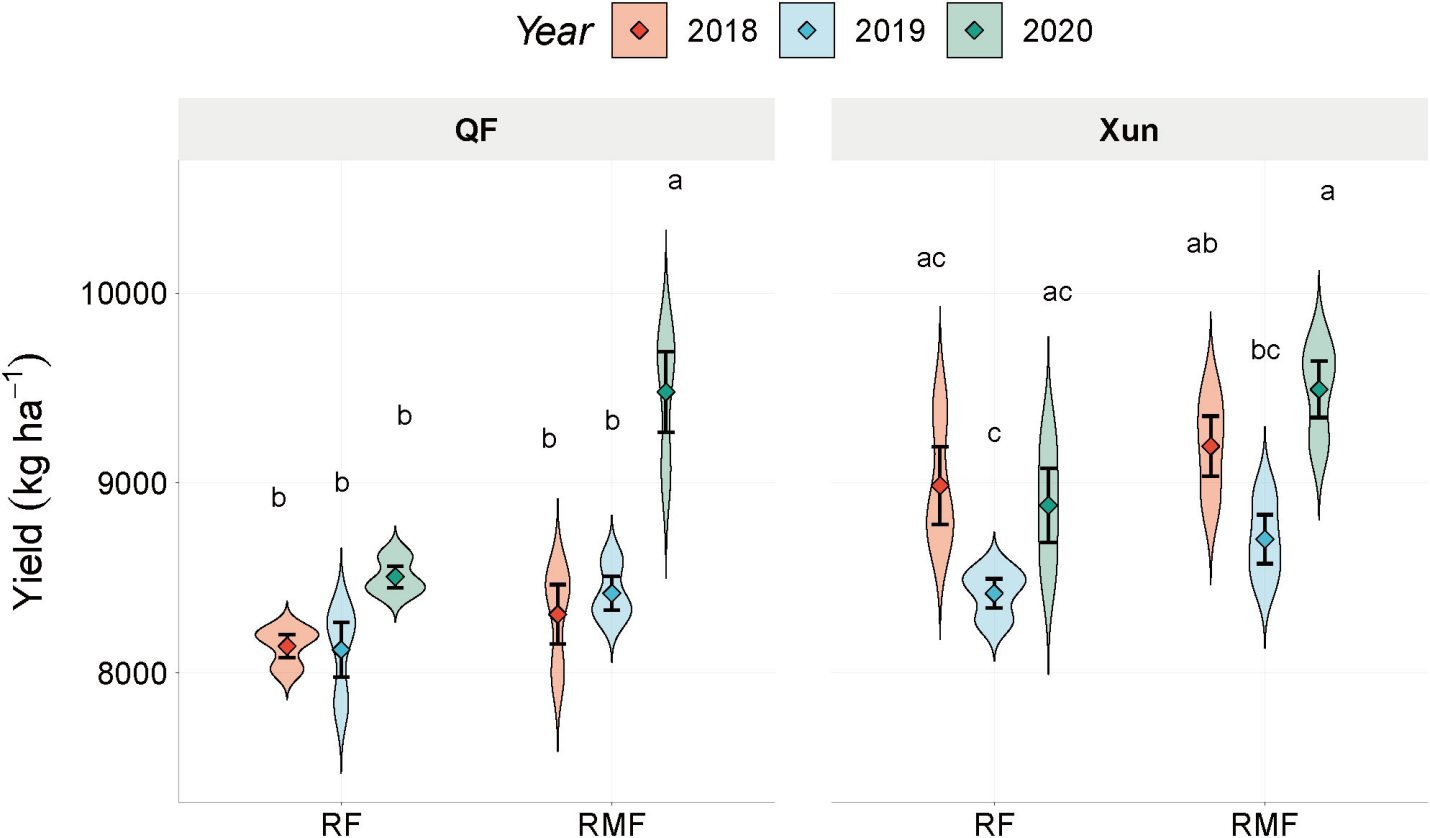
Violin plots showing the distribution of maize yield under the different treatments (CF, MF) at two sites (QF and Xun) over 3 years (2018–2020). Diamonds, mean values with error bars that show the SE. Different letters, statistically significant differences between treatments within each site and year (Tukey’s HSD, *p* < 0.05). CF, chemical fertilizer; MF, microbial fertilizer; QF, Qingfeng Country; Xun, Xun Country.

The use of microbial fertilizer significantly enhanced the SDR (*p* = 0.002). As shown in Figure 1A, under the MF treatment, the SDR increased by 8.4 and 8.6% at QF (*p* < 0.05) and Xun Country (*p* < 0.05), respectively. Similarly, the application of microbial fertilizer significantly increased the maize yield (*p* = 0.001) in the third year, with gains of 11.4% at QF and 6.9% at Xun (*p* < 0.05 at both sites). The yield benefits were cumulative and only significant in year 3 (Figure 2). These findings indicated that the microbial fertilizer conferred no immediate advantage but accumulated over time. It became statistically detectable only after multiple years of application.

The analysis also revealed spatial differences and those related to differences in the microbial community α-diversity indices. The site had a significant effect on the richness of bacterial communities (*p* = 0.011). The application of microbial fertilizer led to a moderate 3.8% increase in richness at QF and a negligible 0.4% change at Xun. However, neither change was statistically significant. The Shannon diversity indices for the bacterial communities remained stable across the treatments and sites (Table 1). The fungal α-diversity were also clearly differentiated spatially, with significantly higher richness at Xun compared to QF (*p* = 0.009). The microbial fertilizer effects were minimal with non-significant increases of 5.8% at QF and 0.9% at Xun.

### Microbial community composition and distribution

The qPCR results (Table 2) indicate the abundance of *B. subtilis* in the soil samples from two sites (Xun and QF) under different treatments (CF and MF). Notably, *B. subtilis* was undetectable (indicated by “—”) in all the CF treatment replicates at site Xun, while measurable quantities were detected under the MF treatment. They ranged from 0.44 to 1.04 copies/g soil. At site QF, *B. subtilis* was detected at low levels in the CF treatment (0.3 × 10^5^ copies/g soil) in one replicate and consistently at higher levels under the MF treatment across all the replicates. These results suggest that the MF treatment promotes a higher abundance of *B. subtilis* compared to the CF treatment. In addition, the sites were variable. *T. harzianum* was not detected above MDL in the bulk-soil samples; therefore, we did not compute site- or treatment-level statistics for this taxon.

**TABLE 2:** The gene abundance of *B. subtilis* in the soil samples from different sites and treatments determined by quantitative PCR (qPCR).

Site	Replicate	CF ( × 10^5^ copies/g soil)	MF ( × 10^5^ copies/g soil)
Xun	1	—	4.4
	2	—	6.1
	3	—	10.4
QF	1	—	1.3
	2	0.3	2.1
	3	—	2.8

DNA extracts were analyzed for the abundance of *B. subtilis* using the primers Bsub_16S_F and Bsub_16S_R by qPCR. QF, Qingfeng Country; Xun, Xun Country; CF, chemical fertilizer; MF, microbial fertilizer; —, no detectable amplification.

**FIGURE 3:**
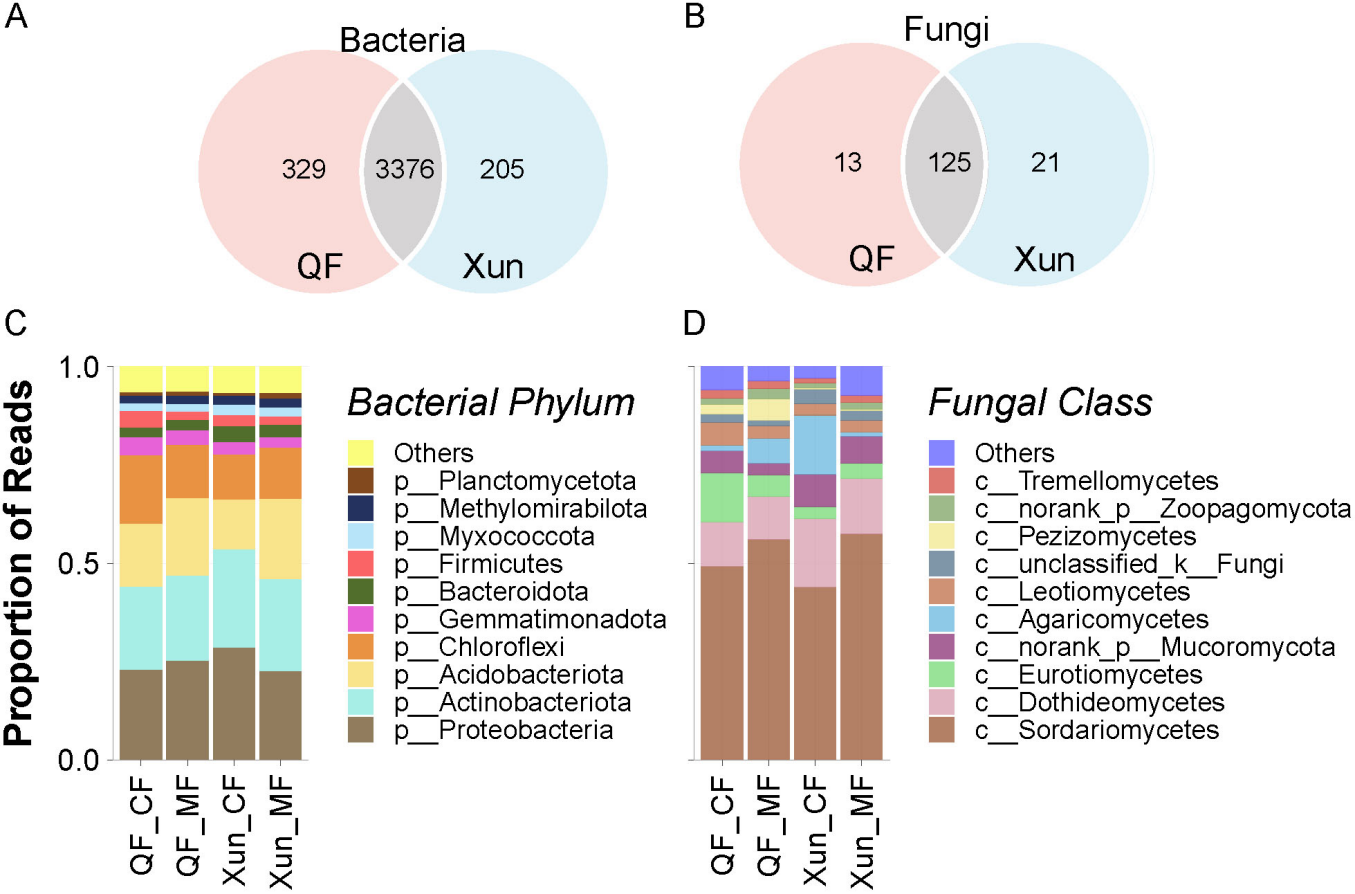
Comparison of the bacterial and fungal community compositions between the QF and Xun groups following treatment with control and microbial fertilizer. Venn diagrams show the number of shared and unique operational taxonomic units (OTUs) between the QF and Xun groups for (A) bacteria and (B) fungi. The overlapping areas represent OTUs shared by both groups, while non-overlapping areas indicate unique OTUs. Stacked bar plots depict the relative abundance of the top 10 bacterial phyla (C) and fungal classes (D) across the four groups: QF_CF, QF_MF, Xun_CF, and Xun_MF. “Others” represents the sum of taxa outside the top 10. Taxonomic groups are color-coded according to the legends. Proportions of reads are shown on the y-axis. CF, chemical fertilizer; MF, microbial fertilizer; QF, Qingfeng Country; Xun, Xun Country.

A total of 3,905 bacterial OTUs and 159 fungal OTUs were detected across all the samples, with most taxa shared between the QF and Xun sites (Figures 3A,B). Proteobacteria, Actinobacteriota, Acidobacteriota, and Chloroflexi were the dominant bacterial phyla across both sites. Despite this high overlap, there were clear significant differences in the relative abundances of certain microbial groups between the two locations. Chloroflexi was 27% relatively more abundant in QF compared to Xun (15.5% vs. 12.2%) (Figure 3C), while Gemmatimonadota was 44% higher in QF than Xun (4.15% vs. 2.89%). In contrast, Bacteroidota was 44% more abundant in Xun than in QF (3.61% vs. 2.50%), and Myxococcota was 31% more abundant in Xun (2.49% vs. 1.90%). Among the fungi, Eurotiomycetes was 161% relatively more abundant in QF compared to Xun (8.97% vs. 3.44%) (Figure 3D), and Pezizomycetes was more than 1,100% higher in QF (3.90% vs. 0.31%). Conversely, unclassified fungi were relatively 73% more abundant in Xun (3.10% vs. 1.79%).

The microbial fertilizer exerted pronounced effects on the dominant bacterial and fungal communities. Relative abundances of Acidobacteriota rose markedly by 22.7% (QF) and 60.8% (Xun), whereas Firmicutes declined significantly by 50.1% and 27.5% in the same sites, respectively. Among fungi, Sordariomycetes showed a substantial increase of 13.7% (QF) and 30.9% (Xun).

### Keystone species analysis

Microbial co-occurrence networks were constructed separately for each site based on a Spearman’s rank correlation analysis. The network was modularized using the cluster_fast_greedy algorithm, and the keystone species were identified based on Zi-Pi metrics.

**FIGURE 4:**
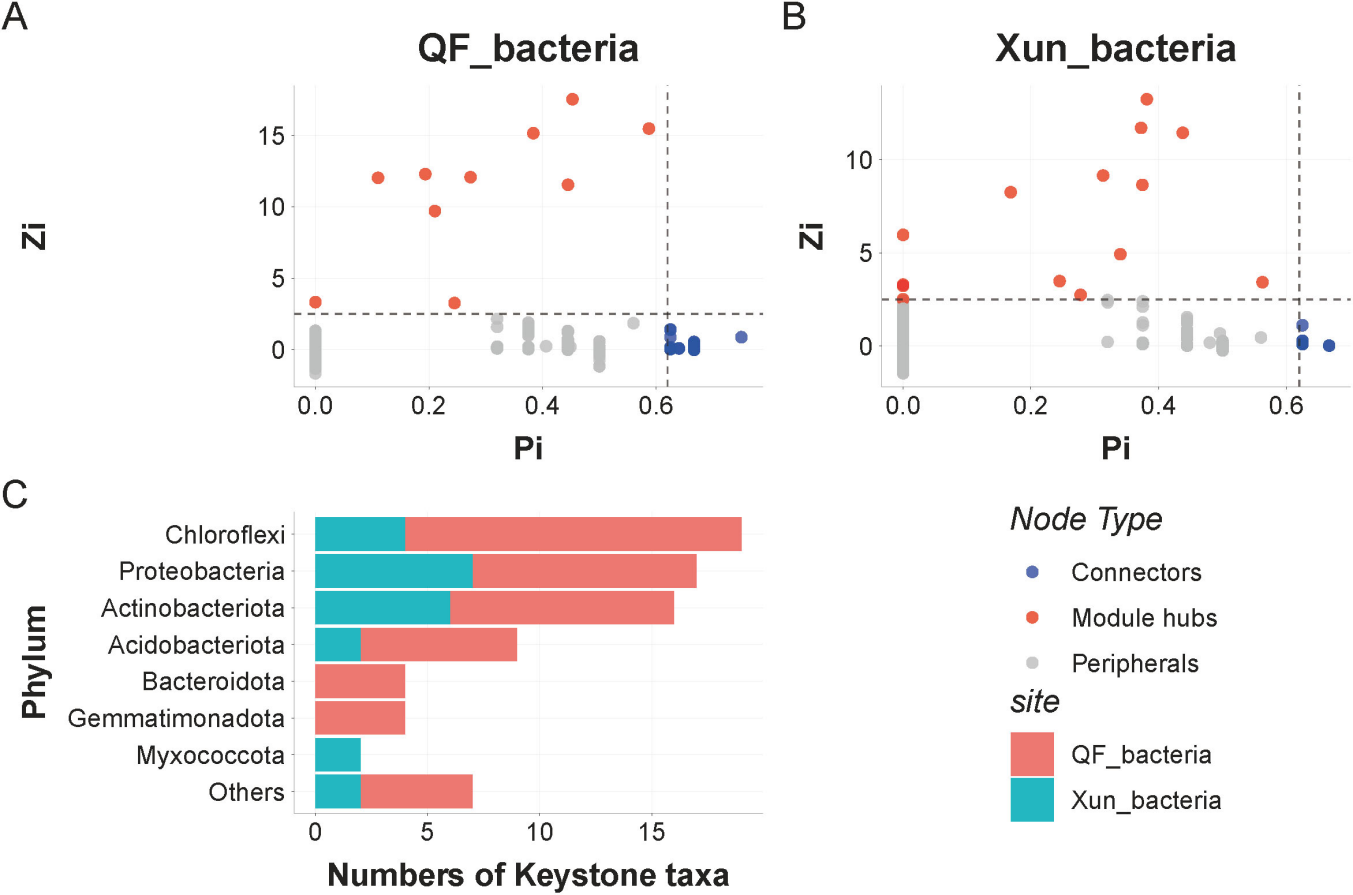
Identification of Keystone Taxa in the Bacterial Co-occurrence Networks Across Sites (A,B) Zi-Pi scatter plots show the topological roles of OTUs in the bacterial networks from Qingfeng Country (QF) and Xun Country (Xun). Module hubs (high Zi) and connectors (high Pi) are designated as keystone taxa. (C) Taxonomic distribution of the keystone OTUs at the phylum level. Module hubs exhibited strong within-module connectivity (e.g., OTU1897 from Qingfeng, Zi = 15.47), while the connectors (e.g., OTU542 from Qingfeng, Pi = 0.625) facilitated cross-module interactions. The major keystone taxa were members of Chloroflexi (19.6%), Proteobacteria (17.5%), and Actinobacteriota (16.5%). OTUs, operational taxonomic units.

The Zi-Pi analysis revealed that the bacterial network at the QF site contained 49 keystone nodes, including six module hubs and 43 connectors (Figures 4A,B), whereas the bacterial network at the Xun site contained 21 keystone nodes. They were composed of 17 module hubs and four connectors. The keystone nodes were predominantly affiliated with the phyla Chloroflexi (19.6%), Proteobacteria (17.5%), and Actinobacteriota (16.5%) (Figure 4C).

Among these, the module hub OTUs; such as OTU1897 from QF, Zi = 15.47; exhibited exceptionally strong within-module connectivity, while the connector OTUs; such as OTU542 from QF, Pi = 0.625; were primarily involved in the inter-module interactions.

### Correlations between the microbial communities and soil factors

**FIGURE 5:**
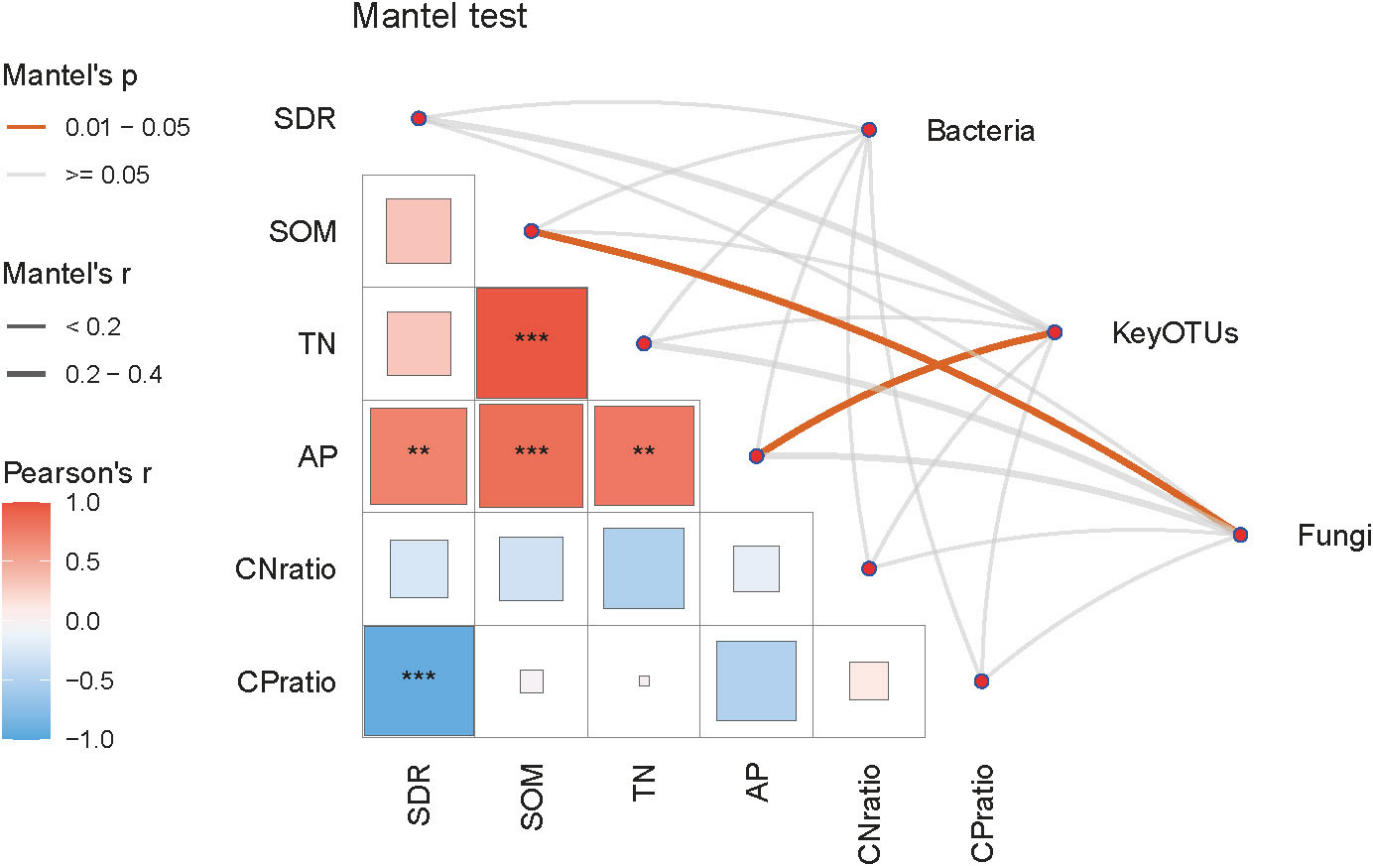
Mantel test and Pearson correlation analyses between the microbial communities and environmental variables. This figure integrates the Mantel test results (right side) and pairwise Pearson correlations (left matrix) to explore the associations between microbial communities—Bacteria, Key operational taxonomic units (KeyOTUs), and Fungi—and key environmental factors, including the straw degradation rate (SDR), soil organic carbon (SOC), total nitrogen (TN), available phosphorus (AP), carbon-to-nitrogen ratio (CNratio), and carbon-to-available phosphorus ratio (CPratio). On the right, the lines that connects the microbial groups to environmental factors indicate Mantel correlations. Line thickness, the strength of Mantel’s r; color, statistical significance (Mantel’s *p* < 0.05 shown in orange; ≥ 0.05 in gray). On the left, the heatmap shows pairwise Pearson correlation coefficients among the environmental variables. Red, positive correlations; blue, negative correlations. Asterisks denote significance levels (**p < 0.01, ***p < 0.001). This combined visualization highlights that the key microbial taxa (KeyOTUs and Fungi) are significantly associated with environmental gradients, particularly the available phosphorus (AP) and soil organic carbon (SOC).

As shown in Figure 5, the Pearson correlation heatmap revealed strong positive relationships among the SOC, TN, and AP (Pearson’s *r* > 0.5, *p* < 0.01) and a significant negative correlation between the SDR and CPratio (*r* < −0.5, *p* < 0.001). Other environmental variable pairs showed generally weak or non-significant correlations.

The Mantel test results showed that the bacterial communities exhibited weak and statistically non-significant correlations with all the environmental parameters (*r* < 0.2, *p* > 0.05). In contrast, the KeyOTUs (key operational taxonomic units) were moderately and significantly associated with the AP (*r* = 0.248, *p* = 0.045) and showed weaker, non-significant associations with the SDR and CPratio. The fungal communities significantly moderately correlated with the SOC (*r* = 0.339, *p* = 0.035) and non-significantly but moderately associated with the TN (*r* = 0.247, *p* = 0.062).

Previous correlation analyses among the environmental factors indicated that the relationships varied. Apart from the significant negative SDR–CP ratio relationship noted above, the SDR had only weak, non-significant correlations with the other soil factors (SOC, TN, AP, and the CN ratio), while the SOC, TN, and AP moderately correlated. This reflected their interconnected roles in the dynamics of soil nutrients. The CNratio and CPratio weakly correlated with the other variables, which suggested that they varied independently.

These findings suggest that while the bacterial communities appeared insensitive to the soil and SDR, the KeyOTUs and fungi responded more strongly to variations in the AP and SOC, respectively. The integration of Mantel and Pearson correlation results supports the interpretation that AP and SOC are the key environmental drivers of the microbial community composition in this system.

### Functional predictions linking community shifts to SDR and AP

**FIGURE 6:**
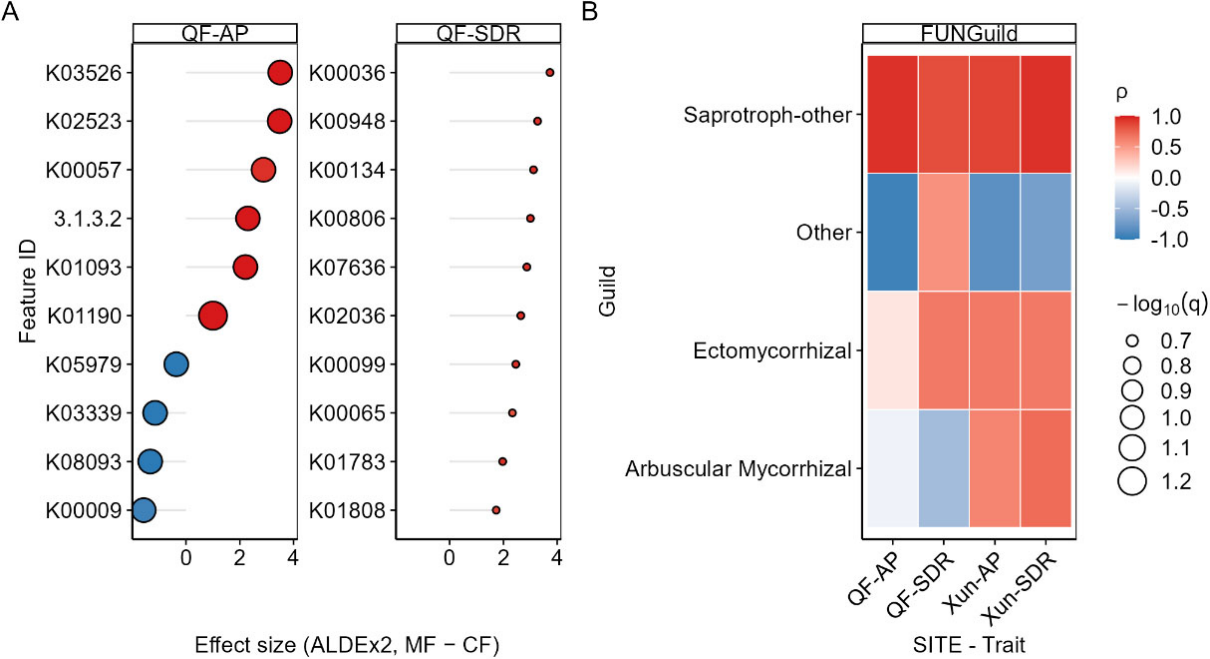
Functional signals linking microbial fertilizer (MF) to straw degradation (SDR) and available phosphorus (AP). Bacterial functional features (PICRUSt2/Tax4Fun) plotted as effect size (MF−CF within site; ALDEx2) versus Spearman correlation with SDR or AP after CLR transformation (A). Points are colored by correlation (ρ) and sized by −log10(q); labels indicate representative features. Right: FUNGuild heatmap showing correlations between fungal guilds and SDR/AP (FDR-adjusted) (B). The enzymes corresponding to Feature TD are listed in Supplementary Data 1, 2.

Across both bacterial predictors (PICRUSt2 and Tax4Fun), MF treatments showed enrichment of functions associated with cellulose/hemicellulose depolymerization and phosphorus turnover relative to CF treatments within sites. Consistent with the observed rise of Sordariomycetes, FUNGuild indicated higher representation of saprotrophic guilds under MF. At the plot level, MF-enriched bacterial functions were positively associated with SDR and AP (FDR-adjusted), and fungal saprotrophic guilds tracked similar gradients. These patterns are summarized in Figure 6, with complete feature lists and statistics in Supplementary Table 1.

### Pathways of microbial fertilizer influence on yield

To further examine the mechanisms by which the microbial fertilizers affect the crop yields, a PLS-PM was constructed using the data from two sampling sites. The model incorporated the following five latent variables: microbial fertilizer treatment, microbial community characteristics, straw degradation rate, soil nutrient status, and crop yield.

**FIGURE 7:**
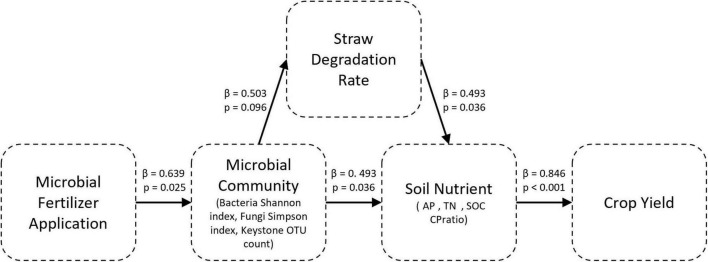
Partial least squares path model (PLS-PM) that illustrates the indirect effects of the application of microbial fertilizer on crop yield. This path diagram depicts the hypothesized causal relationships among these five latent variables: microbial fertilizer treatment, microbial community characteristics (Bacterial Shannon index, Fungal Simpson index, and Keystone OTU count), straw degradation rate (SDR), soil nutrient status (AP, TN, SOC and CPratio), and crop yield (Yield). Arrows, directional paths between variables. They are annotated with the standardized path coefficients (β) and corresponding *p*-values derived from the bootstrap validation (*n* = 500).

As shown in Figure 7, the application of microbial fertilizer significantly influenced the characteristics of microbial community (path coefficient = 0.639, *p* = 0.0254). This finding confirmed that the microbial amendments effectively altered the microbial diversity and abundance in the soil as measured by the bacterial Shannon index, fungal Simpson index, and key functional OTUs. These microbial changes subsequently influenced the straw decomposition rate (β = 0.503, *p* = 0.0959), which reflected a significant effect.

In turn, the microbial communities significantly directly affected the soil nutrient status (β = 0.493, *p* = 0.0362), while the influence of SDR on nutrient status was of comparable magnitude and also statistically significant (β = 0.493, *p* = 0.0364). The soil nutrient status exerted a strong and statistically significant direct influence on the crop yield (β = 0.846, *p* = 0.0005). This finding indicates that the availability of nutrients remains the dominant proximal factor that determines the outcomes of yields.

The model explained 71.5% of the variability in crop yield (*R*^2^ = 0.715), thus demonstrating substantial explanatory power. The bootstrapped total effects confirmed an indirect pathway from the microbial fertilizer treatment to the yield through the microbial community characteristics and improvement in soil nutrients [total effect = 0.40, 95% confidence interval (CI) = 0.143–0.720].

## Discussion

### Regional variability, microbial community dynamics, and yield responses

This study found significant regional differences in the soil physicochemical properties, including the TN, AP, and organic matter, and microbial richness, which confirmed the dominant role of local soil and climatic conditions in shaping baseline soil characteristics. This conclusion is consistent with those of previous studies (Girvan et al., 2003; O’Callaghan et al., 2022). However, while the site effects significantly influenced the levels of soil nutrients, no significant differences were observed in the SDR and maize yield. These findings suggest that the microbial fertilizer performed robustly across contrasting agroecological zones, which reflected the functional resilience of the microbial consortia applied.

The application of microbial fertilizer across both experimental sites significantly increased the SDR by 8.4–8.6% and enhanced the yield of maize by 6.9–11.4%. Notably, the microbial fertilizer treatment led to clearly apparent changes in the soil AP, with significant increases observed at both locations. These findings underline the critical role of microbial amendments in enhancing the bioavailability of P, particularly within agricultural systems that are limited in P, and emphasize their potential to stimulate nutrient cycling and improve crop productivity (Richardson and Simpson, 2011; Shen et al., 2011).

Importantly, the observed improvement in crop yield appears to be primarily mediated by functional shifts in the soil microbial communities rather than by extensive alterations in the overall soil chemical properties. This is consistent with the findings of previous studies that showed that microbial inoculants can effectively promote the mineralization of organic matter by selectively restructuring microbial groups specialized in degrading lignocellulose and mobilizing nutrients (Li et al., 2014; Zhao et al., 2024). Therefore, targeted microbial amendments represent a promising strategy to sustainably enhance the productivity and fertility of the soil through improved P dynamics and microbial functionality.

The use of qPCR to target *B. subtilis* revealed clear evidence of successful colonization by beneficial microbes. This indicates that the inoculated strains were able to effectively establish themselves in the soil and persist in the soil microbiome. In contrast, T. *harzianum* was not quantitatively detected in the bulk soil. This outcome likely reflects its ecological preference for the rhizosphere and root-associated environments, where it thrives but is less recoverable from bulk soil due to limited proliferation in that compartment. The typical soil detection limits, which are around 10^4^ conidia-equivalents per gram, combined with inherent matrix and assay variabilities, can easily mask low or patchy populations of the fungus (Stummer et al., 2020). Moreover, competition from native soil microorganisms, along with fluctuating abiotic conditions such as moisture, pH, and temperature, likely suppress detectable biomass of *T. harzianum* away from the root environment (Khare and Arora, 2015). To effectively distinguish between the synergistic effects and the individual contributions of inoculants in future studies, we recommend targeting the roots and rhizosphere specifically. Utilizing strain-specific TaqMan or ddPCR assays (or employing tagged strains) will enhance detection accuracy. Additionally, monitoring should be conducted shortly after application to capture the initial colonization and activity of the inoculant (Kredics et al., 2018).

The higher abundance of Chloroflexi, which is known for its ability to metabolize C and tolerate environmental stress (Hug et al., 2013; Zheng et al., 2022), at QF (27% greater than Xun) coincided with an improvement in the degradation of straw. This degradation suggests a potential role in C cycling, although mechanistic studies are required to confirm this link. Conversely, the relatively lower yield response observed in Xun may be linked to the greater abundance of Dothideomycetes at this site. These fungi are effective at scavenging nutrients and may act antagonistically toward introduced strains (Absalan et al., 2024; Huang et al., 2024). These findings suggest that priority effects and competition for niches among the indigenous microbial communities can modulate the performance of exogenous inoculants (Debray et al., 2022; Kong et al., 2025). The observed decrease in fungal richness at QF (−7.5%, *p* = 0.077) implies that there could have been competitive exclusion or antagonism by the inoculated taxa, such as *T. harzianum*, thus, underscoring the sensitivity of fungal communities to microbial inputs (Yao et al., 2023; Rigobelo et al., 2024). In contrast, the bacterial diversity remained stable, which is consistent with meta-analyses that suggest that bacterial communities often exhibit functional redundancy and compositional resilience to microbial amendments (Mawarda et al., 2020; Li et al., 2024). The KeyOTUs, such as the copiotrophic taxa, particularly Proteobacteria and Actinobacteriota, further support a shift toward quick growing organisms that cycle nutrients and are associated with improved mineralization, thereby mechanistically explaining the observed improvements in straw decomposition and maize yield (Jenkins et al., 2010; Yao et al., 2017; Gao et al., 2022).

### Mechanistic pathways that link microbial fertilizer to yield

The application of microbial fertilizers significantly improved the yield of maize at both sites, with increases of 11.4% at QF and 6.9% at Xun. These findings highlight the agronomic benefits of biologically enriched inputs. These results are consistent with our hypothesis and several previous studies that document enhanced crop productivity following the application of microbial inoculants (Lugtenberg and Kamilova, 2009; Backer et al., 2018; Gan et al., 2023; Manjunath et al., 2023).

The PLS-PM revealed that the gains in yield were primarily mediated through changes in the structure and function of the microbial community, which subsequently influenced the dynamics of soil nutrients and the turnover of organic matter. This mirrors the concept of plant–microbiome interaction as a multi-step, ecologically assembled process, wherein microbial communities modulate plant nutrition and defense responses through complex biochemical and signaling networks (Trivedi et al., 2020). At the microbiological level, the application of microbial fertilizer significantly reshaped the characteristics of the microbial community (path coefficient = 0.639, *p* = 0.0254), as indicated by shifts in the diversity indices and the enrichment of key operational taxonomic units (KeyOTUs). The enrichment of copiotrophic taxa, such as Proteobacteria and Actinobacteriota, supports a transition toward fast-growing, nutrient-cycling organisms. This is consistent with the global patterns observed across rhizosphere microbiomes where the niches associated with plants selectively recruit highly metabolically versatile microorganisms (Bulgarelli et al., 2015; Levy et al., 2018). These compositional shifts are particularly relevant to the decomposition of organic matter. Compant et al. (2010) emphasized that bacterial traits, such as the secretion of cell wall-degrading enzymes, including cellulases and pectic enzymes, and siderophores are instrumental in the ability of bacteria to compete in the rhizosphere and colonize plants. Similarly, our observed improvements in the SDR, which increased by more than 8% at both sites, probably reflected an increase in the abundance of taxa that produce these types of enzymes. Thus, this confirms the importance of microbial community composition in driving the dynamics of the decomposition of organic matter.

Nutrient mineralization, particularly of P and N, emerged as another key pathway. The AP in the soil increased significantly (15.4% at QF; 19.7% at Xun) following the application of microbial fertilization. This corroborated previous findings that phosphate-solubilizing microorganisms enhance the uptake of P by the plants owing to their production of organic acids (Alori et al., 2017; Pan and Cai, 2023), secretion of enzymes (Richardson and Simpson, 2011) and extrusion of protons (da Silva et al., 2023). In the present study, gene prediction analysis confirmed that phosphatase genes were significantly upregulated following microbial fertilizer application (Figure 6). Although the increases in TN were not statistically significant, the directional trend may be attributed to enhanced ammonification and a reduction in the losses of N: a function that is linked to both free-living and endophytic N fixers (Zhang et al., 2019; Khan et al., 2021; Guo et al., 2023). The increases in SOC (by 8.1% at QF and 3.9% at Xun) further underscore the ecological significance of the turnover of C mediated by microorganisms. Notably, Trivedi et al. (2020) identified the microbial production of osmoprotectants, ACC deaminase, and enzymes that detoxify reactive oxygen species as mechanisms that not only contribute to the tolerance to stress but also to the stabilization of soil organic pools. These soil biochemical enhancements were statistically supported by the Mantel and Pearson correlations. The KeyOTUs were significantly associated with AP (*r* = 0.248, *p* = 0.045), while the fungal community composition moderately correlated with the SOC (*r* = 0.339, *p* = 0.035). These associations are consistent with the findings of Mendes et al., (2011), who observed that rhizosphere community composition could predict suppressiveness and nutrient cycling potential in disease-resistant soils.

Moreover, the PLS-PM model confirmed that the shifts in the microbial community and enhancement in the degradation of straw had significant direct effects on the status of soil nutrients (β = 0.493, *p* < 0.05). In turn, this was the dominant predictor of crop yield (β = 0.846, *p* = 0.0005) and explained 71.5% of the variability (*R*^2^ = 0.715). The PLS-PM model identified two equally important pathways: restructuring of the microbial community (β = 0.493) and acceleration of the decomposition of straw (β = 0.493). Both of these pathways enhanced the availability of soil nutrients and ultimately the yield. These results provide robust support for a biologically mediated, multi-step influence pathway in which microbial fertilizers primarily enhance yield through changes in microbial composition and subsequent improvements in nutrient cycling, rather than by direct mechanisms alone. These findings are consistent with the “holobiont” perspective where plants and their associated microbiota co-function as an ecological unit to enhance the use of resources and resilience to stress (Vandenkoornhuyse et al., 2015). Beyond taxonomic shifts (e.g., Acidobacteriota, Sordariomycetes), functional inference (PICRUSt2/Tax4Fun; FUNGuild) suggests that MF enhances cellulolytic and phosphorus-cycling capacities, consistent with higher SDR and AP and with the PLS-PM pathway; these remain indirect signals, warranting validation via enzyme assays and meta-omics. Our results complement this hypothesis by providing empirical evidence for the role of functionally enriched microbial groups, particularly those that contribute to mineralization, hormonal regulation, and stress mitigation, as critical determinants of plant performance. This study did not assess functional gene expression or microbial activity, which raises questions about the mechanistic roles of specific taxa. Future research could integrate metatranscriptomics to link the community shifts to functional outcomes.

### Implications for sustainable agriculture

This study underscores the critical importance of integrating local microbial ecology and environmental context into the design and application of microbial fertilizers. The region-specific responses observed highlight that inoculant efficacy is strongly influenced by pre-existing microbial assemblages, edaphic characteristics (e.g., soil texture, pH, organic matter), and microclimatic variables such as temperature and moisture. These findings reinforce the need for site-specific strategies informed by ecological assessments at the community level (Kong et al., 2025).

Notably, the present results contribute to global sustainability efforts by demonstrating that microbiome-guided fertilization can accelerate straw decomposition and improve nutrient cycling, particularly phosphorus availability, while maintaining or enhancing maize yields. These benefits align with circular agriculture principles and resource-use efficiency. However, the pronounced site effects observed further emphasize that microbial fertilizers must be customized to local edaphic and climatic conditions, with inoculant selection and dosing tailored to baseline microbiome profiles and soil characteristics.

### Limitations

Our field measurements (yield, SDR, AP) constitute direct experimental evidence for agronomic and biogeochemical responses to MF at two sites, whereas functional predictions and PLS-PM provide a coherent but inferential mechanism linking microbiome restructuring to these responses. We therefore interpret mechanisms cautiously and prioritize enzyme activities (cellulases, phosphatases) and meta-omics as next steps for causal validation. We were unable to robustly quantify T. harzianum in bulk soil (one replicate above MDL; others non-detect), limiting separation of individual vs. synergistic inoculant effects. Functional inferences from 16S/ITS are supportive but indirect. Measurements were concentrated in year 3 and focused on bulk soil, which constrains temporal and spatial resolution. Future work should target root/rhizosphere compartments, apply higher-sensitivity strain-specific assays, and validate functions with enzyme and transcript measurements. These limitations do not alter the observed field responses (higher SDR and yield) but delimit the mechanistic certainty, motivating targeted validation to convert inferential links into causal evidence.

## Conclusion

This study provides field-scale evidence supporting our hypothesis that composite microbial fertilizers enhance maize productivity across two contrasting agro-ecological zones by re-structuring soil microbiomes and improving soil nutrition cycling. Our key findings reveal that these fertilizers simultaneously elevate the levels of available phosphorus and straw degradation rates, creating a soil micro-environment that sustains vigorous crop growth. Structural equation modeling uncovered a cascading mechanism in which the application of microbial fertilizer drives gains in yield through sequential enhancements in microbial diversity, straw decomposition efficiency, and nutrient cycling. Collectively, these enhancements accounted for 71.5% of the variation in observed yield. In summary, these results underscore the strategic value of microbial fertilizers to sustainably intensify cropping systems and emphasize the critical need for region-specific adaptation and microbiome-guided management. Future research should focus on further exploring the specific microbial mechanisms and optimizing the application strategies of microbial fertilizers for different agro-ecological regions.

## Data Availability

The raw sequencing data generated in this study have been deposited in the NCBI Sequence Read Archive (SRA) under BioProject accession number PRJNA1331835. All other data supporting the findings of this study are included in the article and its Supplementary material.
