# Supplementary figures and images for: Microbial fertilizer for improving maize yield, straw decomposition and soil microbiome structure
Source: Front Microbiol. 2025 Dec 15;16:1670118. doi: 10.3389/fmicb.2025.1670118 (PMC12745462; doi:10.3389/fmicb.2025.1670118)

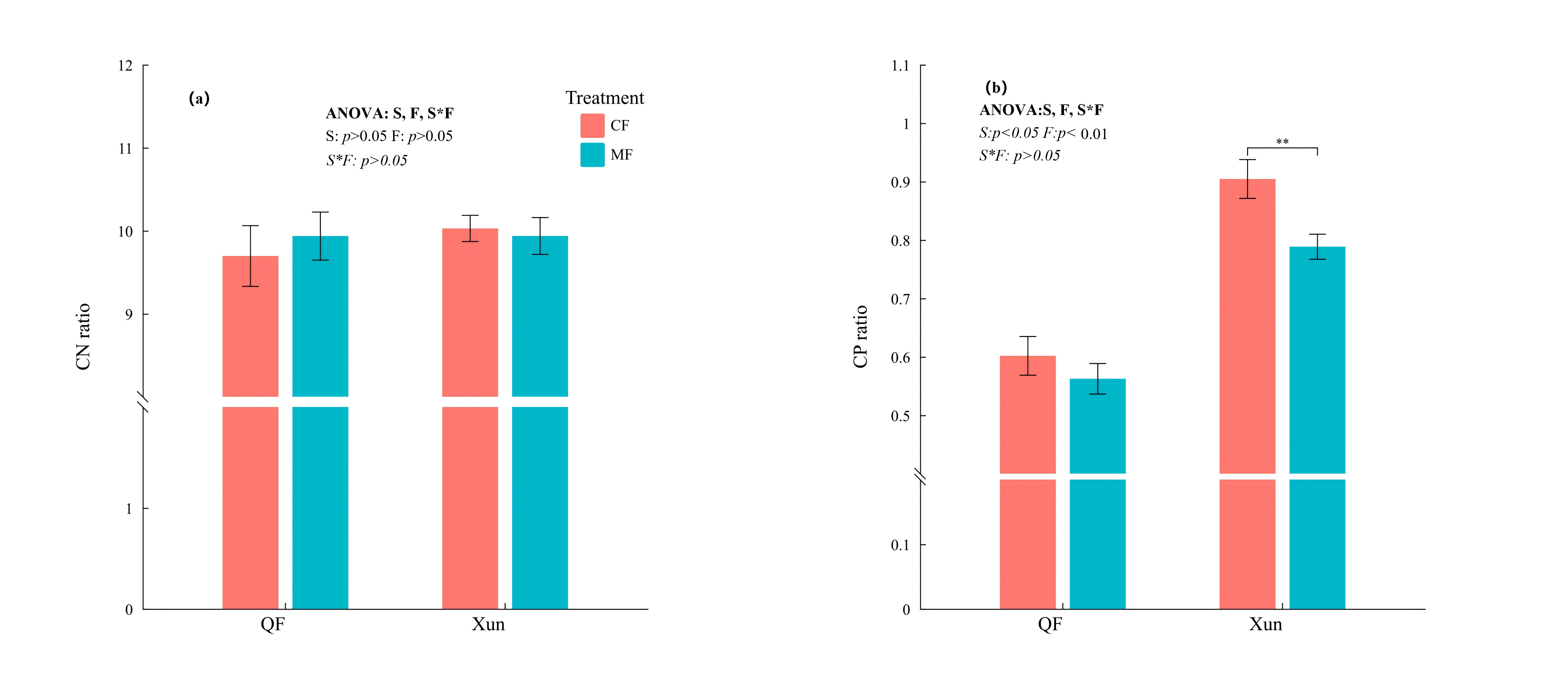

Supplement: Supplementary file 1 [file Image_1.PNG]
